# LINC00467 Promotes Prostate Cancer Progression *via* M2 Macrophage Polarization and the miR-494-3p/STAT3 Axis

**DOI:** 10.3389/fonc.2021.661431

**Published:** 2021-05-19

**Authors:** Hao Jiang, Wen Deng, Ke Zhu, Zhenhao Zeng, Bing Hu, Zhengtao Zhou, An Xie, Cheng Zhang, Bin Fu, Xiaochen Zhou, Gongxian Wang

**Affiliations:** ^1^ Department of Urology, The First Affiliated Hospital of Nanchang University, Nanchang, China; ^2^ Jiangxi Institute of Urology, Nanchang, China

**Keywords:** LINC00467, prostate cancer, M2 macrophage polarization, miR-494-3p, STAT3

## Abstract

**Background:**

The long non-coding RNA LINC00467 plays a vital role in many malignancies. Nevertheless, the role of LINC00467 in prostate carcinoma (PC) is unknown. Herein, we aimed to explore the mechanism by which LINC00467 regulates PC progression.

**Methods:**

We used bioinformatics analyses and RT-qPCR to investigate the expression of LINC00467 in PC tissues and cells. The function of LINC00467 in the progression of PC was confirmed by loss-of-function experiments. PC cell proliferation was assessed by CCK-8 and EdU assays. The cell cycle progression of PC cells was examined by flow cytometry. Moreover, Transwell assays were used to investigate the migration and invasion of PC cells. Western blot assays were used to detect the expression of factors associated with epithelial–mesenchymal transition. The interactions of LINC00467 with prostate cancer progression and M2 macrophage polarization were confirmed by RT-qPCR. The subcellular localization of LINC00467 was investigated *via* the fractionation of nuclear and cytoplasmic RNA. Bioinformatics data analysis was used to predict the correlation of LINC00467 expression with miR-494-3p expression. LINC00467/miR-494-3p/STAT3 interactions were identified by using a dual-luciferase reporter system. Finally, the influence of LINC00467 expression on PC progression was investigated with an *in vivo* nude mouse model of tumorigenesis.

**Results:**

We established that LINC00467 expression was upregulated in PC tissues and cells. Downregulated LINC00467 expression inhibited PC cell growth, cell cycle progression, migration, and invasion. Downregulated LINC00467 expression similarly inhibited PC cell migration *via* M2 macrophage polarization. Western blot analysis showed that LINC00467 could regulate the STAT3 pathway. We established that LINC00467 is mainly localized to the cytoplasm. Bioinformatics analysis and rescue experiments indicated that LINC00467 promotes PC progression *via* the miR-494-3p/STAT3 axis. Downregulated LINC00467 expression was also able to suppress PC tumor growth *in vivo*.

**Conclusions:**

Our study reveals that LINC00467 promotes prostate cancer progression *via* M2 macrophage polarization and the miR-494-3p/STAT3 axis.

## Introduction

Prostate cancer (PC) is one of the most common tumors of the urinary system. According to the latest statistics from the National Center for Health Statistics (NCHS), the incidence and mortality of prostate cancer in the United States are predicted to be 191,930 and 33,330, respectively, in 2020 (1). Despite the rapid development of approaches for the diagnosis and treatment of prostate cancer, the poor treatment effect and high prevalence rate are still serious challenges in the clinic (2). Therefore, the identification of new potential biomarkers and therapeutic targets is crucial for the improvement of alternative therapies.

Recently, a large number of ncRNAs (non-coding RNAs) have been identified, and these ncRNAs are important components of the complex regulatory network of the body. LncRNAs (long non-coding RNAs), a class of RNAs with lengths greater than 200 nts, do not encode proteins but regulate the expression of genes at the RNA level in a variety of ways (3). Researchers have recognized that functional lncRNAs participate in a variety of physiological and pathological processes (4, 5), particularly oncogenesis, because these lncRNAs can affect gene expression by sponging microRNAs and mRNAs in various tumor types (6). For example, PCA3 (7), PCGEM1 (8) and PlncRNA-1 (9) are known to be overexpressed in prostate cancer and to promote cancer progression.

In the present study, we showed that LINC00467 expression was upregulated in human prostate cancer tissues and cell lines. However, the function of LINC00467 in PC, as well as the underlying mechanism, remains unknown. Therefore, we investigated the function of LINC00467 in PC progression and its underlying mechanism, providing a new theoretical basis for the treatment of PC.

## Materials and Methods

### Prostate Cancer Cell Lines and Clinical Tissues

The human prostate cancer (PC) cell lines VCaP, LNCaP, 22RV1, PC3, and DU145 and the normal human prostate epithelial cell lines HrPEC and RWPE-1 were purchased from American Type Culture Collection (ATCC, www.atcc.rog, USA). LNCaP, 22RV1, DU145 and THP-1 cells were cultured in RPMI-1640 (Gibco, USA), PC3 cells were cultured in F12K medium (Gibco), VCaP and RWPE1 cells were cultured in DMEM (Gibco, USA), and HrPEC cells were cultured in ATCC prostatic primary epithelial cell culture medium. Ten percent fetal bovine serum (FBS, Gibco) and 1% penicillin streptomycin (Solarbio, China) were added to the medium, and the cells were cultured in a humidified environment at 37°C and 5% CO_2_. THP-1 monocytes were treated with 100 nM phorbol 12-myristate 13-acetate (PMA) (Sigma-Aldrich, USA) for 24 h to induce their differentiation into macrophages. To obtain conditioned media (CM) from the PC cells, the indicated PC cells were grown to 70–80% confluence, washed three times with FBS-free medium, and cultured in fresh FBS-free medium for another 72 h. Next, the supernatants were filtered through 0.22-µm filters and collected for use as CM.

From September 2017 to June 2019, we collected clinical tissues from 22 PC patients undergoing prostatectomy in the urology department of the First Affiliated Hospital of Nanchang University. Prostate tumor tissues and paired adjacent normal tissues were collected. After collection, all the samples were immediately stored in a −80°C freezer until further treatment.

### Cell Transfection

LINC00467 siRNA, miR-494-3p inhibitor and miR-494-3p mimics were designed and synthesized by RiboBio (Guangzhou, China). The sequences of the siRNAs were as follows: siLINC00467-1#: 5’-ACACTAAGTTCAGACTCAT-3’; siLINC00467-2#: 5’-TCAGACTCATGAAACCAAT-3’. The siRNAs were transfected into THP-1, 22RV1 and DU145 cells using Lipofectamine 3000 according to the manufacturer’s instructions. The sequence of siLINC00467-2# was used for lentivirus transfection. shLINC00467 and shNC (negative control) vectors were transfected with lentivirus (Hanbio, Shanghai, China). The lentiviral vectors were added to 22RV1 and DU145 culture media. After 72 h, 22RV1 and DU145 cells carrying the constructs were selected using puromycin (selection concentration, 6 µg/ml; maintenance concentration, 2 µg/ml).

### RNA Extraction and Real-Time Quantitative RT-PCR (RT-qPCR) Analysis

Total RNA was extracted and reverse transcribed into cDNA using the RNeasy Mini kit (Qiagen, USA) and the First-Strand cDNA Synthesis kit (Qiagen, USA) according to the manufacturer’s instructions. RT-qPCR was performed using a SYBR Real-Time PCR kit (Qiagen, USA) under the following conditions: 95°C for 2 min, followed by 40 cycles of 95°C for 5 s and 60°C for 10 s. Relative fold expression was calculated using the 2−ΔΔCt method. Each analysis was performed in triplicate. In addition, β-actin was used as an internal reference gene. The primers used in the study are listed in **Table 1**.

**Table 1:** Primers used for RT-qPCR.

Genes	Forward (5′-3′)	Reverse (5′-3′)
β-actin	CATGTACGTTGCTATCCAGGC	CTCCTTAATGTCACGCACGAT
LINC00467	TTCGGTCCGGTTGAGGTTGT	AAACCTCCCTGCCATGTTGGA
STAT3	ACCAGCAGTATAGCCGCTTC	GCCACAATCCGGGCAATCT
U6	AAAGCAAATCATCGGACGACC	GGGGTCGTTGATGGCAACA
MALAT1	GCCAAATTGAGACAATTTCAGC	CGAATTCAGGGTGAGGAAGTA
CD86	CTGCTCATCTATACACGGTTACC	GGAAACGTCGTACAGTTCTGTG
INOS	TCATCCGCTATGCTGGCTAC	CCCGAAACCACTCGTATTTGG
CD163	TTTGTCAACTTGAGTCCCTTCAC	TCCCGCTACACTTGTTTTCAC
MRC1	GGGTTGCTATCACTCTCTATGC	TTTCTTGTCTGTTGCCGTAGTT
ARG1	TGGACAGACTAGGAATTGGCA	CCAGTCCGTCAACATCAAAACT
IL10	TCAAGGCGCATGTGAACTCC	GATGTCAAACTCACTCATGGCT
VEGF	AGGGCAGAATCATCACGAAGT	AGGGTCTCGATTGGATGGCA

### CCK8 Assay

Cell Counting Kit 8 (CCK-8) assays were used to analyse the proliferation of lentivirus-transfected DU145 and 22RV1 cells. After transfection, the cells in the logarithmic stage of growth (DU145 cells, 6 × 10^3^ cells/well; 22RV1 cells, 5 × 10^4^ cells/well) were seeded in a 96-well culture plate. After 1 day, 2 days, 3 days, and 4 days, 100 µl RPMI-1640 medium and 10 µl CCK8 solution were added to each well. After the cells were incubated at 37°C for 2 h, the OD value was measured at 450 nm by a microplate system. Each experiment was repeated three times.

### Ethynyl Deoxyuridine (EdU) Assay

A 5-ethynyl-2’-deoxyuridine (EdU) detection kit (RiboBio, Guangzhou, China) was used to assess the proliferation of lentivirus-transfected DU145 and 22RV1 cells according to the manufacturer’s guidelines. Transfected DU145 and 22RV1 cells in the logarithmic stage of growth (DU145 cells, 1 × 10^4^ cells/well; 22RV1 cells, 3 × 10^4^ cells/well) were seeded in a 96-well culture plate. After the cells were cultured for 24 h, the cells were treated with 50 µM EdU for 2 h at 37°C, fixed with 4% paraformaldehyde for 30 min, incubated with 2 mg/ml glycin for 5 min, incubated with PBS containing 0.5% Triton X-100 for 10 min, stained with 1× Apollo staining reaction solution for 30 min, incubated with PBS containing 0.5% Triton X-100 for 10 min, and finally incubated with 100 μl of 1× Hoechst 33342 for 30 min. The percentage of EdU-positive cells was examined using a fluorescence microscope. Each experiment was repeated three times.

### Transwell Assay

For the cell migration and invasion assays, the transfected cells (DU145 cells, 4 × 104 cells/well; 22RV1 cells, 1 × 105 cells/well) were cultured in an upper Transwell chamber coated with FBS-free RPMI-1640 medium (without Matrigel added; 8-µm pores, Corning, USA), and 600 µl RPMI-1640 medium containing 20% foetal bovine serum was added to the lower chamber. The 22RV1 cells were incubated for 48 h, and the DU145 cells were incubated for 24 h. The cells in the lower chamber were fixed with 4% paraformaldehyde for 20 min and stained crystal violet staining solution for 30 min, and the excess cells in the upper chamber were removed. Then, the chambers were dried. Three fields were randomly selected for each sample to capture the penetrating cells and take pictures. All the assays were conducted three independent times.

### Cell Cycle Assay

DU145 and 22RV1 cells were harvested 48 h after transfection and washed with ice-cold PBS solution. Then, the cells were fixed with 75% ethanol overnight at 4°C, resuspended in propidium iodide (PI)/RNase A solution (5 μg/ml PI and 100 mg/ml RNase A) and incubated for 15 min at room temperature in the dark. Then, a flow cytometer (Millipore, Guava) was utilized to analyse cell cycle progression. All the assays were conducted three independent times.

### Subcellular Fractionation

Nuclear and cytoplasmic RNA were extracted using the NE-PER nuclear and cytoplasm extraction reagent kit (Thermo Scientific, USA) following the manufacturer’s protocol. Then, the relative RNA levels in the nucleus and cytoplasm were measured by RT-qPCR. MALAT1 served as a positive nuclear control, U6 served as a nuclear control transcript, and β-actin served as a cytoplasmic control.

### Western Blotting

Transfected DU145 and 22RV1 cells were collected and lysed on ice with RIPA lysis buffer (Applygen, Beijing, China) containing protease inhibitors and phosphatase inhibitors for 30 min and centrifuged at 12,000*g* for 15 min, and the supernatants were collected. The extracted proteins were quantified by a BCA Protein Assay Kit (Trans, China). Then, the total cellular proteins were subjected to SD-PAGE (10%) for Western blot analysis. After transferring the proteins to polyvinylidene difluoride (PVDF) membranes, the membranes were blocked with 5% BSA in Tris-buffered saline with Tween for 60 min and incubated overnight at 4°C on a rocker with the following primary antibodies: β-Actin (CST, 4970, 1:1,000), STAT3 (CST, 9139, 1:1,000), pSTAT3 (CST, 9145, 1:1,000), E-cadherin (CST, 3195, 1:1,000), N-cadherin(CST, 13116, 1:1,000) and Vimentin (CST, 5741, 1:1,000). After washing three times with TBST for 10 min, the membranes were incubated with a horseradish peroxidase (HRP)-labeled goat anti-rabbit (CST, 7074, 1:4,000) or anti-mouse (CST, 7076, 1:4,000) secondary antibody for 1 h at room temperature (RT) and washed six times with TBST for 5 min. The protein bands were visualized by enhanced chemiluminescence (ECL).

### Tumor Xenograft Model in Nude Mice

DU145 cells transfected with shLINC00467 or shNC were subcutaneously inoculated with Matrigel into 6-week-old male BALB/c nude mice. The tumor weights and volumes were measured once a week, and of the tumor volumes were calculated as follows: V = 3.14/6Ddd (D = tumor length, d = tumor width). Finally, the mice were sacrificed, the tumors were removed, and the tumor weights were recorded.

### Hematoxylin Eosin (HE) Staining

Xenograft tissues from nude mice were paraffin embedded, sliced, and dried at 65°C. After drying, the sections were dewaxed with xylene for 20 min, dehydrated with gradient ethanol of 100, 95, 85 and 75% for 5 min, and washed with tap water for 2 min. Then, the sections were stained with haematoxylin for 10 min and washed with tap water for 2 min, hydrochloric acid and ethanol for 10 s, and ammonia antiblue for 10 s. The sections were stained with eosin for 3 min and then rinsed with distilled water. The sections were dehydrated with gradient ethanol and then cleared with xylene. The HE staining results were observed and photographed under an optical microscope.

### Immunohistochemistry

Xenograft tissues from nude mice were paraffin embedded, sliced, and dried at 65°C. After drying, the sections were dewaxed with xylene for 20 min and dehydrated with gradient ethanol solutions of 100, 95, 85 and 75% for 5 min. Then, the sections were incubated with citric acid buffer for 10 min for antigen repair, with peroxidase for 15 min, and with 10% goat serum for 30 min. The primary antibody was added and incubated at 4 °C overnight. The secondary antibody was added and incubated at room temperature for 30 min, and diaminobenzidine (DAB) staining was used to adjust the chrominance under the microscope. The sections were washed with tap water for 5 min, stained with haematoxylin for 5 min, differentiated with 1% hydrochloric acid and ethanol for 5 s, stained with reverse blue for 5 s with ammonia, sealed with neutral glue, and observed and photographed with light microscopy.

### Statistical Analysis

All the data are expressed as the means ± SD with at least three replicates in each group. Statistical analysis was performed to determine the significance of the difference between groups using ANOVA or Student’s t test. All the statistical analyses were performed using GraphPad/Prism software for Windows. Differences were considered to be statistically significant when *p <*0.05.

## Results

### High Expression of LINC00467 in PC Tissues and Cells

**Figure 1:**
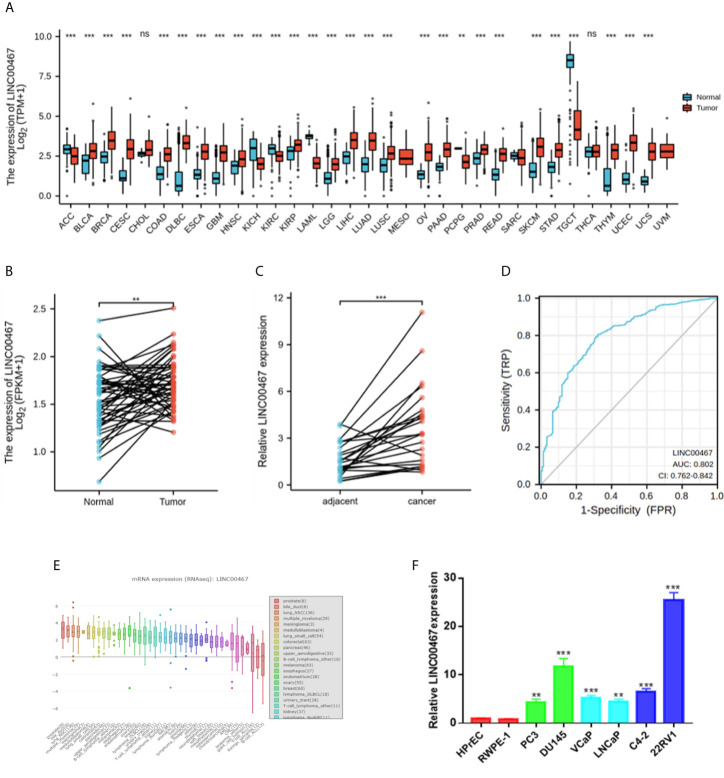
LINC00467 expression was markedly higher in human prostate cancer tissues and cell lines. **(A)** TCGA and GTEx databases showed LINC00467 expression in most human cancers. **(B)** TCGA and GTEx database analysis showing LINC00467 expression in 49 pairs of prostate cancer samples. **(C)** RT-qPCR analysis showing LINC00467 expression in 22 pairs of prostate cancer samples. **(D)** CCLE database showed LINC00467 expression in different cancer cell lines. **(E)** RT-qPCR analysis showing LINC00467 expression in prostate cancer cell lines and nonmalignant cell lines. **(F)** ROC curves indicating the sensitivity and specificity of LINC00467 expression in prostate cancer samples. **p < 0.01, ***p < 0.001; the data are presented as the means ± SD, n = 3. ns, not statistically significant (p>0.05).

First, the RNA-seq data obtained from the GTEx and TCGA databases were analysed. The results showed that there were higher LINC00467 levels in most cancer samples (**Figure 1A**). Then, we analysed 49 pairs of PC tissues obtained from the GTEx and TCGA databases. The level of LINC00467 was markedly higher in PC tissues than in neighboring nonmalignant tissues (**Figure 1B**). RT-qPCR analysis showed that LINC00467 was overexpressed in 24 PC tissues compared with neighbouring nonmalignant tissues (**Figure 1C**). ROC curves revealed the sensitivity and specificity of LINC00467 expression in prostate cancer samples (**Figure 1D**). Furthermore, we analysed the RNA-seq data obtained from the Cancer Cell Line Encyclopedia (CCLE) database and found that LINC00467 was overexpressed in PC cell lines compared with other cancer cell lines (**Figure 1E**). RT-qPCR analysis demonstrated that LINC00467 was highly expressed in PC cell lines compared with nonmalignant prostate cell lines (**Figure 1F**).

### Effects of LINC00467 Downregulation on Prostate Cancer Cell Proliferation, Cell Cycle Progression, Migration and Infiltration

**Figure 2:**
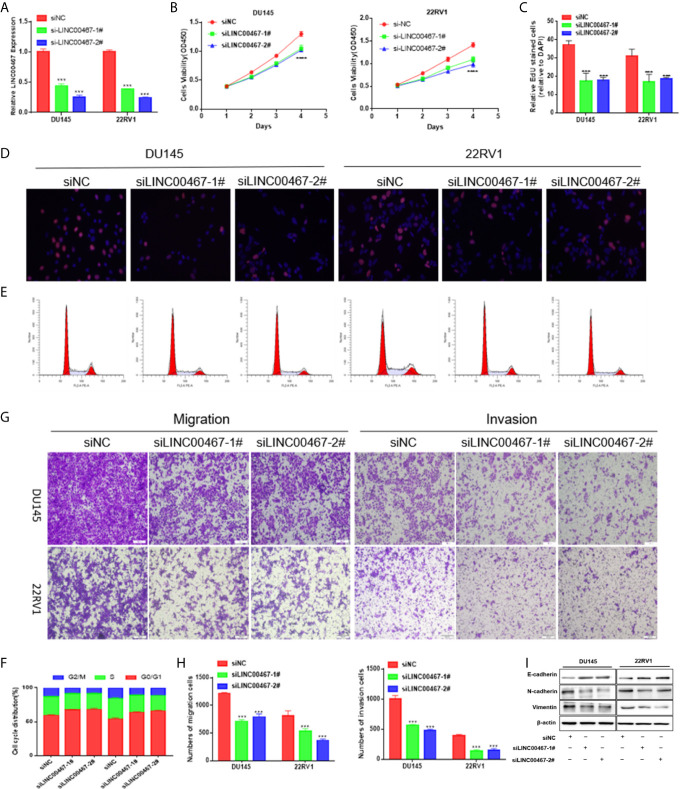
LINC00467 downregulation inhibits cell growth, cell cycle progression, migration, and infiltration. **(A)** RT-qPCR assessment of LINC00467 expression levels in DU145 and 22RV1 cells after transfection with siLINC00467 or the negative control. **(B, D)** CCK-8 and EdU assay data indicating proliferation of DU145 and 22RV1 cells transfected with siLINC00467 or the negative control. **(E, F)** Cell cycle progression results indicating that DU145 and 22RV1 cells were arrested in the G0/G1 phase after treatment with siLINC00467. **(G, H)** Cell migration and invasion results indicating that the downregulation of LINC00467 expression inhibits the migration and invasion of DU145 and 22RV1 cells. **(I)** Western blot analysis showed that the EMT protein was decreased after treatment with siLINC00467. ***p < 0.001, ****p < 0.0001; the data are presented as the means ± SD, n = 3.

To investigate the physiological effects of LINC00467, LINC00467 expression was silenced in the DU45 and 22RV1 cell lines (**Figure 2A**). With CCK-8 and EdU assays, we found that knockdown of LINC00467 expression inhibited the proliferation of DU145 and 22RV1 cells (**Figures 2B–D**). Abnormal cell cycle progression may lead to decreased cell proliferation. Therefore, we measured the effect of LINC00467 knockdown on cell cycle progression. Based on flow cytometry analysis, we found that LINC00467 knockdown resulted in increased DU45 and 22RV1 cell numbers in the G0/G1 phase and decreased cell numbers in the S and G2/M phase (**Figures 2E, F**). The Transwell assay data demonstrated that LINC00467 silencing inhibited the migration and infiltration of DU45 and 22RV1 cells (**Figures 2G, H**). Epithelial–mesenchymal transition (EMT) is a basic process by which epithelial cells lose their epithelial characteristics and transform into mesenchymal cells, thereby reducing intercellular adhesion and increasing cell motility (10). Western blotting analysis showed that knockdown of LINC00467 expression inhibited the expression of EMT-related proteins (**Figure 2I**), suggesting that LINC00467 may plays a role in the process of EMT.

### LINC00467 Downregulation Inhibits PC Cell Migration and Invasion by Decreasing M2 Macrophage Polarization *Via* the STAT3 Pathway

**Figure 3:**
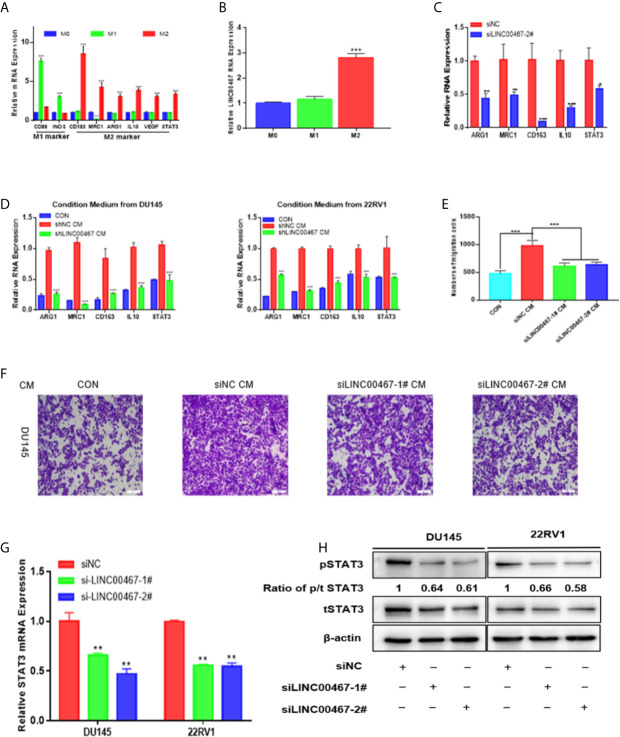
LINC00467 downregulation inhibits PC cell migration and invasion by decreasing M2 macrophage polarization *via* the STAT3 pathway. **(A)** RT-qPCR was used to assess the expression of M1 markers and M2 markers after treatment with LPS/INF-γ or IL-4/IL-13. **(B)** The expression of LINC00467 was higher in M2 macrophages. **(C)** M2 biomarker expression was markedly decreased in the LINC00467-silenced M2 macrophage group. **(D)** Conditioned medium derived from LINC00467-knockdown cells further decreased the expression of M2 biomarkers and LINC00467 in macrophages. **(E, F)** Downregulation of LINC00467 expression in macrophages inhibited the migration of DU145 cells. **(G)** RT-qPCR showing that STAT3 mRNA expression was decreased in LINC00467-knockdown PC cells. **(H)** Western blot indicating that the downregulation of LINC00467 expression decreased the expression of tSTAT3, pSTAT3 and the ratio of p/t STAT3 in PC cells. *p < 0.05, **p < 0.01, ***p < 0.001; the data are presented as the means ± SD, n = 3.

Previous investigations documented that TAMs (tumor-associated macrophages) with an M2-like phenotype are the most predominant immune-associated cells in the TME (tumor microenvironment) and are involved in tumor development by inducing angiogenesis, metastasis, and immune escape. To investigate whether LINC00467 participates in M2 polarization, we assessed the expression of LINC00467, M1 biomarkers, and M2 biomarkers in IL-4/IL-13-treated M2 macrophages, unpolarized macrophages, and LPS/IFN-γ-treated M1 macrophages. Consequently, the expression levels of M1-associated genes (iNOS and CD86) were markedly higher in M1 macrophages, while those of M2-associated genes (CD163, MRC-1, ARG1 and IL10) were markedly higher in M2 macrophages (**Figure 3A**); these results suggested successful monocyte polarization. In addition, there was higher LINC00467 content in M2 macrophages than in M1 macrophages (**Figure 3B**), suggesting that LINC00467 may be related to the polarization of M2 macrophages. After treatment with PMA for 24 h, we transfected THP-1 cells with siNC (negative control) or siLINC00467, added IL-4 and IL-13 and incubated the cells for 24 h to stimulate polarization toward the M2 phenotype. Consequently, the expression of M2 signature genes was markedly decreased in the siLINC00467 groups (**Figure 3C**). Moreover, the supernatants from LINC00467-knockdown DU145 and 22RV1 cells exhibited lower M2 biomarker levels than those from control DU145 and 22RV1 cells, as shown in **Figure 3D**. Then, we explored the mechanism responsible for the crosstalk between macrophages and PC cells. To further assess whether LINC00467-mediated M2 macrophages enhance tumor development, macrophages were treated with the supernatants obtained from LINC00467-silenced or control cells. Then, we collected the conditioned medium from treated macrophages and used it to treat DU145 cells. The results showed that the macrophages treated with supernatants from LINC00467-silenced cells markedly inhibited cell migration, as indicated in **Figures 3E, F**. Furthermore, a large number of studies have shown that M2 macrophage polarization promotes PC progression (11–13). Taken together, these results suggested that LINC00467 could enhance polarization toward the M2 phenotype and promote the tumor-enhancing role of these M2 macrophages.

In our investigation of the molecular mechanism by which LINC00467 promotes PC cell development, we found that LINC00467 can induce M2 macrophage polarization and that M2 macrophage polarization can activate the STAT3 pathway. Thus, we hypothesized that LINC00467 can influence the expression of pSTAT3 and tSTAT3 in PC cells. RT-qPCR analysis showed that downregulated LINC00467 expression decreased the mRNA expression of STAT3 in PC cells (**Figure 3G**). Western blot indicating that the downregulation of LINC00467 expression decreased the expression of tSTAT3 (total STAT3), pSTAT3 (phosphorylated STAT3) and the ratio of p/t STAT3 (phosphorylated STAT3/total STAT3) in PC cells (**Figure 3H**), suggesting that LINC00467 could directly inhibit pSTAT3 expression and indirectly inhibited pSTAT3 expression through tSTAT3.

### miR-494-3p Acts as a Medium of LINC00467 and STAT3 in PC

**Figure 4:**
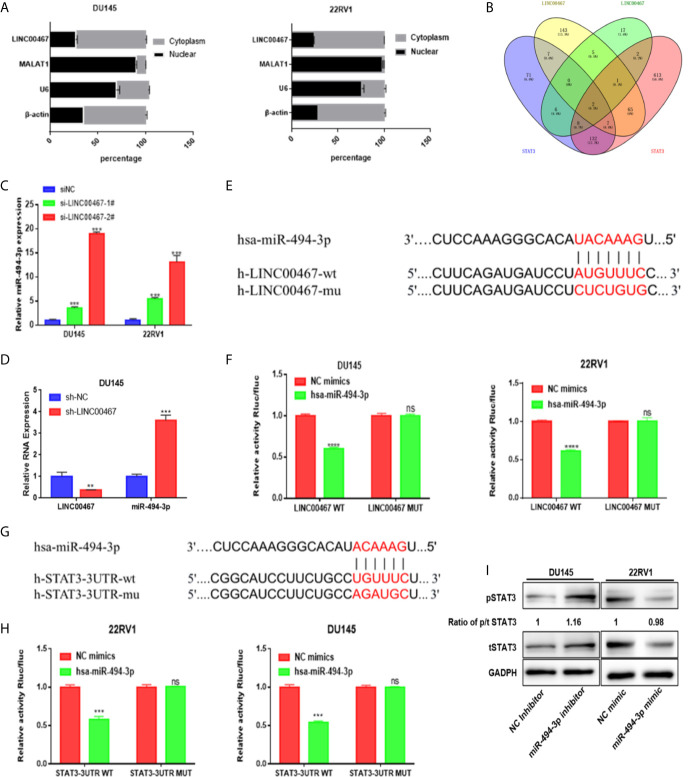
miR-494-3p acts as a medium of LINC00467 and STAT3 in PC. **(A)** Subcellular fractionation indicating that LINC00467 is located mainly in the cytoplasm. **(B)** Venn analysis showing the bioinformatics prediction of miRNAs that target LINC00467 and STAT3. **(C, D)** RT-qPCR analysis showing that miR-494-3p expression is upregulated in LINC00467-knockdown DU145 and 22RV1 cells. **(E, F)** Dual-luciferase reporter assay confirmed the interaction between miR-494-3p and LINC00467. **(G, H)** A dual-luciferase reporter system confirmed the interaction between miR-494-3p and STAT3. **(I)** Western blotting analysis showing that the miR-494-3p inhibitor increased the expression of pSTAT3 and tSTAT3, while the miR-494-3p mimic decreased the expression of pSTAT3 and tSTAT3. **p < 0.01, ***p < 0.001; the data are presented as the means ± SD, n = 3. ns, not statistically significant (p>0.05).

A series of lncRNAs have been found to be able to competitively bind to miRNAs as endogenous RNAs, thus preventing miRNAs from binding to target genes (14). Given that LINC00467 was mainly located in the cytoplasm (**Figure 4A**), we evaluated whether LINC00467 could serve as miRNAs sponge or ceRNAs (competing endogenous RNAs).

The LncBase and starBase databases were used to predict the interaction between miRNA and LINC00467, and starBase and TargetScan were used to predict the interaction between miRNA and STAT3. Venn diagram analysis was used to combine all the predicted miRNAs, and only two miRNAs (miR-1252 and miR-494-3p) could bind to LINC00467 and STAT3 (**Figure 4B**). RT-qPCR was used to assess miR-494-3p expression in LINC00467-knockdown DU145 and 22RV1 cells (**Figures 4C, D**). We found that the binding sites of miR-494-3p were complementary to the sequence of LINC00467 and the 3’UTR of STAT3. A luciferase reporter assay was used to evaluate the crosstalk between miR-494-3p and STAT3 and between miR-494-3p and LINC00467. In the cells transfected with the miR-494-3p mimic, the luciferase activities of the wild-type LINC00467 and STAT3 vectors were markedly lower than that of the reporter vector mutated at the LINC00467 and STAT3 binding sites (**Figures 4E–H**). Then, we used Western blotting to determine how miR-494-3p affects the STAT3 pathway. The miR-494-3p mimic inhibited the expression of pSTAT3 and tSTAT3 in 22RV1 cells, and the miR-494-3p inhibitor enhanced the expression of pSTAT3 and tSTAT3 in DU145 cells (**Figure 4I**). But miR-494-3p could only regulate the expression of tSTAT3 and pSTAT3, not change the ratio of p/t STAT3, indicating that miR-494-3p only indirectly inhibited phosphorylated STAT3 expression through total STAT3. These results suggest that miR-494-3p directly targets STAT3 in PC cells.

### miR-494-3p Inhibition Affects Prostate Cancer Cell Proliferation and Infiltration by Targeting STAT3

**Figure 5:**
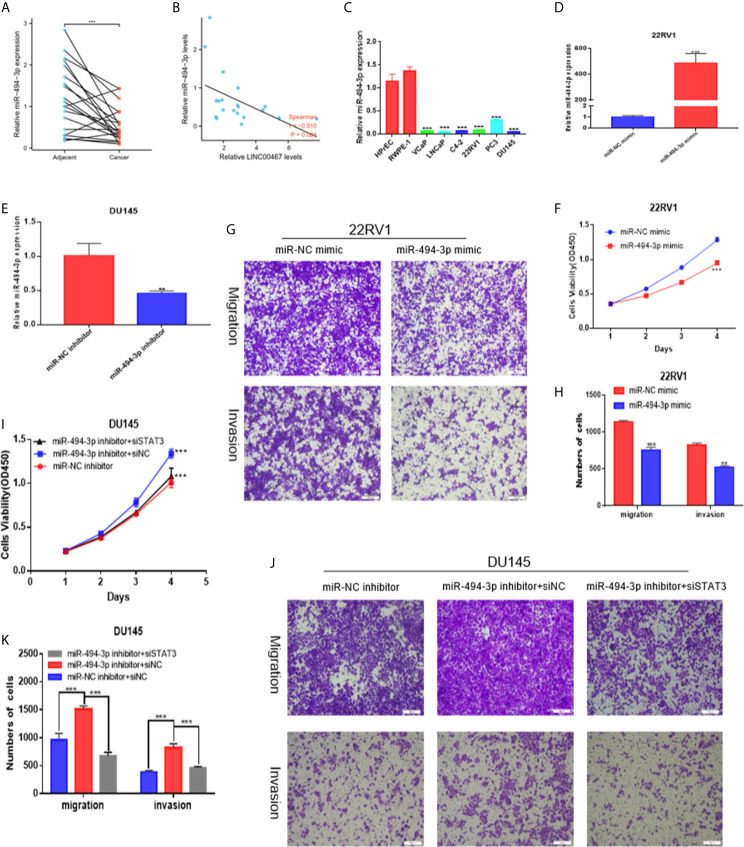
miR-494-3p inhibits prostate cancer progression by targeting STAT3. **(A, C)** RT-qPCR indicating that miR-494-3p expression was downregulated in PC tissues and cell lines compared to nonmalignant tissues and cell lines. **(B)** A negative correlation between the expression of LINC00467 and miR-494-3p was showed using Spearman correlation analysis. **(D, E)** RT-qPCR was used to assess the efficiency of the miR-494-3p inhibitor and mimic in PC cells. **(F–H)** CCK-8 and Transwell assays showing that the miR-494-3p mimic repressed the proliferation, migration and invasion of 22RV1 cells. **(I–K)** CCK-8 and Transwell assays indicating that the miR-494-3p inhibitor promoted the proliferation, migration and invasion of DU145 cells, which could be reversed by siSTAT3. **p < 0.01, ***p < 0.001; the data are presented as the means ± SD, n = 3.

To investigate the expression level of miR-494-3p in PC and its effect on the malignant phenotype, we first analysed the expression of miR-494-3p in 20 pairs of PC tissues and PC cell lines using RT-qPCR and found that miR-494-3p expression was downregulated in PC tissues and PC cell lines compared to normal tissues and cell lines (**Figures 5A, C** **)**. miR-494-3p expression was negatively correlated with LINC00467 expression (**Figure 5B**). Then, we transfected 22RV1 cells with a miRNA mimic (**Figure 5D**) and DU145 cells with a miRNA inhibitor (**Figure 5E**). Elevated expression of miR-494-3p markedly inhibited cell viability, migration, and infiltration (**Figures 5F–H**). Knockdown of miR-494-3p expression markedly promoted cell viability, migration and infiltration, but these events were alleviated by siSTAT3 (**Figures 5I–K**). These data demonstrated that miR-494-3p inhibited PC development by targeting STAT3 *in vitro*.

### LINC00467 Regulates the Proliferation, Migration and Invasion of Prostate Cancer Cells by Modulating the miR-494-3p/STAT3 Pathway

**Figure 6:**
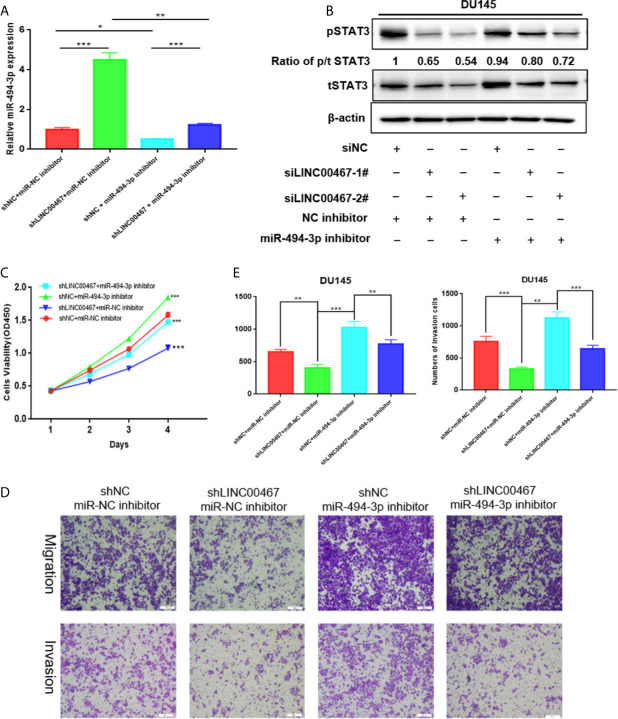
LINC00467 enhances prostate cancer progression by targeting the miR-494-3p/STAT3 axis. **(A)** qPCR analysis indicating miR-494-3p expression. **(C–E)** CCK-8 and Transwell assays showing that shLINC00467 inhibited the proliferation, migration and infiltration abilities of DU145 and 22RV1 cells, and when co-transfected with miR-494-3p inhibitor, the effect was reversed. **(B)** Western blotting analysis indicating that miR-494-3p could partially increase the protein levels of tSTAT3 and pSTAT3, which were decreased by LINC00467, but not rescue the ratio of p/t STAT3. *p < 0.05, **p < 0.01, ***p < 0.001; the data are presented as the means ± SD, n = 3.

To further investigate whether LINC00467 functions by modulating the miR-494-3p/STAT3 cascade, we conducted rescue experiments in DU145 and 22RV1 cells by transfecting these cells with a miR-494-3p inhibitor. We first verified the transfection efficiency by analysing the miR-494-3p levels (**Figure 6A**) and then performed CCK-8 and Transwell assays. LINC00467 gene knockout reduced the proliferation, migration and invasion of DU145 cells. However, miR-494-3p inhibition increased the proliferation, migration, and invasion of prostate cancer cells (**Figures 6C–E**). These findings suggest that LINC00467 plays a tumor-promoting role in prostate cancer, while miR-494-3p plays an inhibitory role in prostate cancer. The downregulated of LINC00467 reduced the expression of tSTAT3, pSTAT3 and the ratio of p/t STAT3, but miR-494-3p could only rescue the expression of tSTAT3 and pSTAT3, suggesting that LINC00467 may regulate the expression of STAT3 pathway through other pathways (**Figure 6B**). Hence, LINC00467 plays a role by regulating the miR-494-3p/STAT3 signaling pathway.

### Downregulation of LINC00467 Expression Represses Tumor Growth in Nude Mouse Xenografts

**Figure 7:**
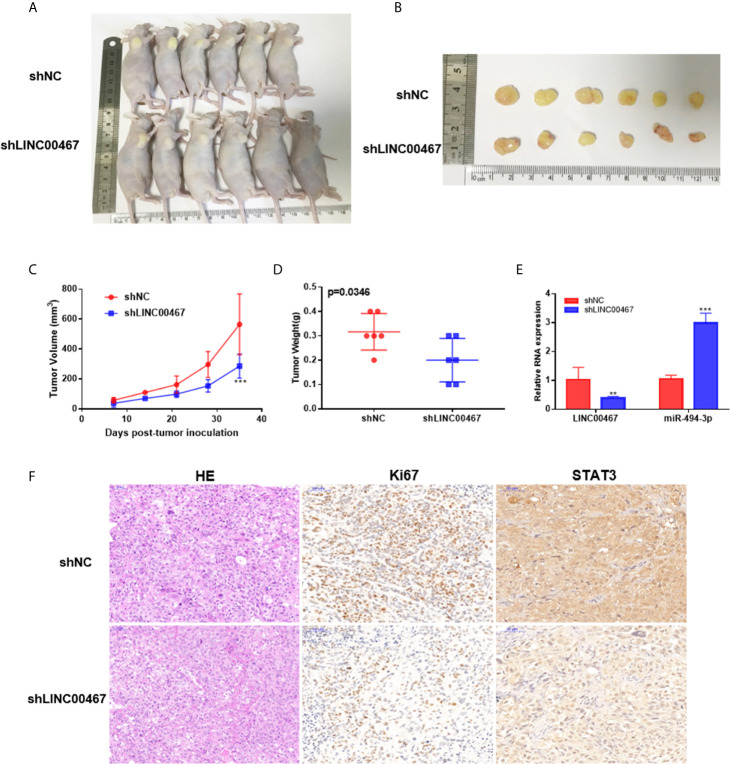
LINC00467 promotes the growth of prostate cancer tumors *in vivo*. **(A)** Subcutaneous administration of DU145 cells stably transfected with shLINC00467 or control vector into nude mice. (n = six per group). **(B)** Image illustrating xenograft tumor formation in nude mice. **(C, D)** The rate of tumor growth and weight were markedly reduced in the shLINC00467 group. **(E)** RT-qPCR analysis indicating the LINC00467 and miR-494-3p levels in tumor tissues. **(F)** HE and IHC staining indicating the expression of Ki67 and STAT3 in tumor tissues. **p < 0.01, ***p < 0.001; the data are presented as the means ± SD, n = 3.

To investigate whether LINC00467 participates in the progression of prostate cancer *in vivo*, we used lentivirus to construct a stable strain LINC00467-knockdown DU145 cells and implanted these cells in nude mice to induce subcutaneous tumor formation. The results showed that LINC00467 knockdown inhibited tumor growth and weight, as well as volume, compared with the lentivirus-mediated stable control vector (**Figures 7A–D**). Therefore, we believe that downregulation of LINC00467 expression can inhibit the formation of xenograft tumors in nude mice. RT-qPCR showed that silencing LINC00467 expression could promote miR-494-3p expression and inhibit STAT3 expression (**Figures 7E, F**). These results suggest that LINC00467 plays a role through its functions in the LINC00467/miR-494-3p/STAT3 axis.

## Discussion

Despite significant advances in diagnosis and treatment, prostate cancer remains the primary cause of morbidity and mortality among men in the United States. Therefore, exploring new therapeutic markers has important clinical significance for prostate cancer patients. In our study, the GTEx and TCGA databases screening and analysis showed that LINC00467 was overexpressed in most human cancers, including prostate cancer. Then analyzed 49 pairs of PC tissues from the GTEx and TCGA databases and qRT-PCR analysis detected 24 pairs of PC tissues, results showed that the level of LINC00467 was markedly higher in PC tissues than in neighbouring nonmalignant tissues, suggesting that LINC00467 may function as a cancer-promoting gene.

LINC00467 expression is aberrantly upregulated in multiple tumor tissues and can enhance cell proliferation in neuroblastoma (15), lung cancer (16), and glioma cells (17). LINC00467 expression is closely associated with worse prognosis of these tumors. The first study about LINC00467 revealed its carcinogenic functions in neuroblastoma; LINC00467 silencing decreased the proliferation of tumor cells but enhanced apoptosis, indicating that LINC00467 functions as a tumor repressor (15). A previous study revealed that LINC00467 promoted proliferation and invasion, inhibited apoptosis, and contributed to axitinib resistance in hepatocellular carcinoma through miR-509-3p/PDGFRA (18). Another study demonstrated that LINC00467 enhances the progression of non-small cell lung cancer through the AKT signaling cascade, and TDG-induced acetylation is the pivotal factor that modulates the expression of LINC00467 (16). Hence, to investigate the physiological effects of LINC00467, downregulated LINC00467 expression inhibited PC cell growth, cell cycle progression, migration, and invasion.

Recently, attention has been focused on the importance of the tumor microenvironment in the progression of tumors. The tumor microenvironment is a complex system consisting of cancer cells, cancer-related fibroblasts, and immune inflammatory cells (19). The crosstalk of cancer cells with TAMs (tumor-associated macrophages), one of the most predominant immune cells in many solid cancers, was linked to cancer progression, drugs resistance and worse prognosis in individuals with cancer (20). On the basis of their biological characteristics, macrophages are grouped into two main phenotypes, namely, proinflammatory (M1) and anti-inflammatory (M2) macrophages. Numerous studies have documented that TAMs are recognized as M2-like macrophages, which are strongly linked to the progression of cancer (21–24). Herein, we established that the LINC00467 levels were markedly higher in M2-like macrophages than in M1-like macrophages and unpolarized macrophages. In addition, LINC00467 knockdown markedly inhibited the expression of M2-like macrophage biomarkers, indicating that LINC00467 promotes the polarization of M2 macrophages. Conditioned medium from prostate cancer cells affected macrophage polarization, reflecting the existence of a transferred mediator. Downregulation of LINC00467 expression in macrophages decreased the migration of PC cells.

A previous study showed that macrophage polarization can increase the migration of pancreatic cancer cells *via* activated STAT3 (25). STAT3 plays a core role in the progression of multiple cancers. STAT3 is a pivotal oncogenic protein that is constitutively activated in PC (26). After activation by phosphorylation, STAT3 translocates into the nucleus and modulates the transcription of numerous genes that participate in antiapoptosis, proliferation, and metastasis processes. In our study, we found that downregulated LINC00467 can inhibit the activated of STAT3 pathway.

The biological roles of lncRNAs primarily depend on their subcellular localization. Growing research evidence has documented that lncRNAs located in the cytoplasm are involved in gene regulation at the posttranscriptional level, such as by functioning as ceRNAs and protecting target mRNAs from suppression (14, 27). Our cell cytoplasmic/nuclear fractionation data demonstrated that LINC00467 was preferentially localized to the cytoplasm, suggesting that it sponges miRNAs.

Therefore, we used the LncBase, StarBase and TargetScan databases to predict the miRNAs that can interact with LINC00467 and STAT3. Venn analysis showed that only two miRNAs were identified, miR-494-3p and miR-1252. miRNA-494-3p has been documented to inhibit the proliferation, infiltration, and migration of prostate cancer cells (28). Herein, miR-494-3p expression was reported to be downregulated in prostate cancer, suggesting a tumor suppressive effect of miR-494-3p, which was consistent with previous studies. The miR-494-3p inhibitor increased tSTAT3 expression and increased the pSTAT3 levels, which confirmed that miR-494-3p regulated the progression of prostate cancer by targeting STAT3. Rescue experiments confirmed that STAT3 is involved in the regulation of miR-494-3p to inhibit the progression of prostate cancer, and miR-494-3p participates in the modulation of LINC00467 to promote the progression of prostate cancer. miR-494-3p is considered to be negatively regulated by LINC00467 *in vivo* and *in vitro*. All the evidence showed that LINC00467 regulated STAT3 to promote the progression of prostate cancer by sponging miR-494-3p.

## Conclusions

Our data revealed that LINC00467 was overexpressed in prostate cancer tissues and cell lines. LINC00467 served as an oncogene in prostate cancer progression. LINC00467 promoted M2 macrophage polarization. The repression of LINC00467 inhibited proliferation and infiltration by regulating the miR-494-3p/STAT3 cascade. Hence, inhibiting LINC00467 could be a prospective therapeutic target for patients with early stage prostate cancer.

## Data Availability Statement

The raw data supporting the conclusions of this article will be made available by the authors, without undue reservation.

## Ethics Statement

The studies involving human participants were reviewed and approved by the First Affiliated Hospital of Nanchang University. The patients/participants provided their written informed consent to participate in this study. The animal study was reviewed and approved by the First Affiliated Hospital of Nanchang University.

## Author Contributions

HJ and XZ performed the experiments and generated data. HJ, WD, KZ, ZZe, BH, ZZh, CZ, and AX analyzed data. XZ, GW, and BF designed the experiments. HJ, XZ, and GW wrote the manuscript. All authors contributed to the article and approved the submitted version.

## Funding

This work was supported by the National Natural Science Foundation of P.R. China (Grant Nos. 82060467 and 81760457) and the Natural Science Foundation of Jiangxi (Grant No. S2017ZRZDB0212).

## Conflict of Interest

The authors declare that the research was conducted in the absence of any commercial or financial relationships that could be construed as a potential conflict of interest.
